# Genomic benchmarks: a collection of datasets for genomic sequence classification

**DOI:** 10.1186/s12863-023-01123-8

**Published:** 2023-05-01

**Authors:** Katarína Grešová, Vlastimil Martinek, David Čechák, Petr Šimeček, Panagiotis Alexiou

**Affiliations:** 1 Centre for Molecular Medicine, Central European Institute of Technology (CEITEC), Masaryk University, Brno, Czechia; 2 National Centre for Biomolecular Research, Faculty of Science, Masaryk University, Brno, Czechia

**Keywords:** Genomics, Dataset, Benchmark, Deep learning, Convolutional neural network

## Abstract

**Background:**

Recently, deep neural networks have been successfully applied in many biological fields. In 2020, a deep learning model AlphaFold won the protein folding competition with predicted structures within the error tolerance of experimental methods. However, this solution to the most prominent bioinformatic challenge of the past 50 years has been possible only thanks to a carefully curated benchmark of experimentally predicted protein structures. In Genomics, we have similar challenges (annotation of genomes and identification of functional elements) but currently, we lack benchmarks similar to protein folding competition.

**Results:**

Here we present a collection of curated and easily accessible sequence classification datasets in the field of genomics. The proposed collection is based on a combination of novel datasets constructed from the mining of publicly available databases and existing datasets obtained from published articles. The collection currently contains nine datasets that focus on regulatory elements (promoters, enhancers, open chromatin region) from three model organisms: human, mouse, and roundworm. A simple convolution neural network is also included in a repository and can be used as a baseline model. Benchmarks and the baseline model are distributed as the Python package ‘genomic-benchmarks’, and the code is available at https://github.com/ML-Bioinfo-CEITEC/genomic_benchmarks.

**Conclusions:**

Deep learning techniques revolutionized many biological fields but mainly thanks to the carefully curated benchmarks. For the field of Genomics, we propose a collection of benchmark datasets for the classification of genomic sequences with an interface for the most commonly used deep learning libraries, implementation of the simple neural network and a training framework that can be used as a starting point for future research. The main aim of this effort is to create a repository for shared datasets that will make machine learning for genomics more comparable and reproducible while reducing the overhead of researchers who want to enter the field, leading to healthy competition and new discoveries.

## Background

Recently, deep neural networks have been successfully applied to identify functional elements in the genomes of humans and other organisms, such as promoters [1], enhancers [2], transcription factor binding sites [3], and others. Neural network models have been shown to be capable of predicting histone accessibility [4], RNA-protein binding [5], and accurately identify short non-coding RNA loci within the genomic background [6].

However, deep neural network models are highly dependent on large amounts of high-quality training data [7]. Comparing the quality of various deep learning models can be challenging, as the authors often use different datasets for evaluation, and quality metrics can be heavily influenced by data preprocessing techniques and other technical differences [8].

Many computational fields have developed established benchmarks, for example, SQuAD for question answering [9], IMDB Sentiment for text classification [10], and ImageNet for image recognition [11]. Benchmarks are crucial in driving innovation. The annual competition for object identification [12] catalyzed the boom in AI, leading in just seven years to models that exceed human capabilities.

In biology, a great challenge over the past 50 years has been *the protein folding problem*. To compare different protein folding algorithms, the community introduced the Critical Assessment of protein Structure Prediction (CASP) [13] challenge benchmark that provides research groups with the opportunity to objectively test their methods. In 2021, AlphaFold [14] won this competition producing predicted structures within the error tolerance of experimental methods. This carefully curated benchmark led to the solution of the most prominent bioinformatic challenge of the past 50 years.

In Genomics, we have similar challenges in annotation of genomes and identification and classification of functional elements, but currently we lack benchmarks similar to CASP. Practically, machine learning tasks in Genomics commonly involve the classification of genomic sequences into several categories and/or contrasting them to a genomic background (a negative set). For example, a well-studied question in Genomics is the prediction of enhancer loci on a genome. For this question, the benchmark situation is highly fragmented. As an example, [15] proposed a benchmark dataset based on the chromatin state from multiple cell lines. Both enhancer and non-enhancer sequences were retrieved from experimental chromatin information. The CD-HIT software [16] was used to filter similar sequences, and the benchmark dataset was made available as a pdf file. However, information stored in a pdf file is suitable for human communication, but computers cannot easily extract data from these files. Despite not being easily machine readable, it was used by many subsequent publications ([2, 17–26] or [27]) as a gold standard for enhancer prediction, highlighting the need for benchmark datasets in this field. Other common sources of enhancer data are the VISTA Enhancer Browser [28], the FANTOM5 [29], the ENCODE project [30], and the Roadmap Epigenomics Project [31] which provide a wealth of positive samples but no negatives. A researcher would need to implement their own method of negative selection, thus introducing individual selection biases to the samples.

Another highly studied question in Genomics is the prediction of promoters. Benchmark situation in this field has its own problems. For example, [32] extracted positive samples from EPD [33] and the non-promoter sequences were randomly extracted from coding regions and non-coding regions, and used as two negative sets. This method for creating a negative set is not an established one. Other authors used only coding sequences or only non-coding sequences as a negative set [34] or combined coding and non-coding sequences as a one negative set [35–37]. Even [32] are already pointing to the problem of missing benchmarks and reproducibility, saying that it is difficult to compare their results with other published results due to differences in data and experimental protocol. Several years later, [38] created their own dataset and reported similar problems. They were unable to compare the results with other published tools because the datasets were derived from different sources, used different proprocessing procedures, or were not made available at all.

In this paper, we propose a collection of benchmark datasets for the classification of genomic sequences, focusing on ease of use for machine learning purposes. The datasets are distributed as a Python package ’genomic-benchmarks’ that is available on GitHub^1^ and distributed through The Python Package Index (PyPI)^2^. The package provides an interface that allows the user to easily work with the benchmarks using Python. Included are utilities for data processing, cleaning procedures, and summary reporting. Additionally, it contains functions that make training a neural network classifier easier, such as PyTorch [39] and TensorFlow [40] data loaders and notebooks containing basic deep learning architectures that can be used as templates for prototyping new methods. Importantly, every dataset presented here comes with an associated notebook that fully reproduces the dataset generation process, to ensure transparency and reproducibility of benchmark generation in the future.

## Construction and content

### Overview of Datasets

**Fig. 1:**
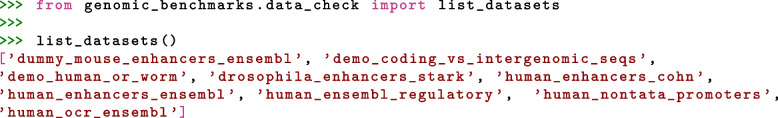
Python code for listing all available dataset in the Genomic benchmarks package

The currently selected datasets are divided into three categories. There is a group of datasets focused on human regulatory functional elements, either produced from mining the Ensembl database, or from published datasets used in multiple articles. For promoters, we have imported human non-TATA promoters [41]. For enhancers, we used human enhancers from [42] paper, Ensembl human enhancers from the FANTOM5 Project [29] and drosophila enhancer [43]. We have also included open chromatin regions and multiclass datasets composed of three regulatory elements (enhancers, promoters, and open chromatin regions), both constructed from the Ensembl regulatory build [44]. The second category consists of ’demo’ datasets that were computationally generated for this project, and focus on classification of genomic sequences between different species or types of transcripts (protein coding vs non-coding). Finally, the third category ’dummy’ has a single small dataset which can be used for quick prototyping of methods due to its small size. From the point of view of the model organism, our datasets include primarily human data, but also mouse (*Mus musculus*), and roundworm (*Caenorhabditis elegans*) and fruit fly (*Drosophila melanogaster*). An overview of available datasets is given in Table 1 and simple code for listing all currently available datasets in Fig. 1. Additional examples of usage can be found in the project’s README (dataset info, downloading the dataset, getting dataset loader), TensorFlow/PyTorch workflows in ‘notebooks‘ folder and finally ‘experiments‘ folder contains papermill runs for each combination of a dataset and a framework.

**Table 1:** Description of datasets in genomic benchmark package. Several pieces of information are provided about each dataset: a) *Name* is unique identification of dataset in genomic benchmark package b) *# of sequences* is combined count of all sequences from all classes c) *# of classes* is count of all classes in a dataset d) *Class ratio* is a ratio between number of sequences in a biggest class and number of sequences in a smallest class e) *Median length* is computed for all sequences from all classes in a dataset f) *Standard deviation* is also computed for all sequences from all classes in a dataset

Name	# of sequences	# of classes	Class ratio	Median length	Standard deviation
dummy_mouse_enhancers_ensembl	1210	2	1.0	2381	984.4
demo_coding_vs_intergenomic_seqs	100000	2	1.0	200	0.0
demo_human_or_worm	100000	2	1.0	200	0.0
drosophila_enhancers_stark	6914	2	1.0	2142	285.5
human_enhancers_cohn	27791	2	1.0	500	0.0
human_enhancers_ensembl	154842	2	1.0	269	122.6
human_ensembl_regulatory	289061	3	1.2	401	184.3
human_nontata_promoters	36131	2	1.2	251	0.0
human_ocr_ensembl	174756	2	1.0	315	108.1

The *Human enhancers Cohn* dataset was adapted from [42]. Enhancers are genomic regulatory functional elements that can be bound by specific DNA binding proteins so as to regulate the transcription of a particular gene. Unlike promoters, enhancers do not need to be in a close proximity to the affected gene, and may be up to several million bases away, making their detection a difficult task.

The *Drosophila enhancers Stark* dataset was adapted from [43]. These enhancers were experimentally validated and we excluded the weak ones. Original coordinates referred to the dm3 [45] assembly of the D. melanogaster genome. We used pyliftover^3^ tool to map coordinates to the dm6 assembly [46]. Negative sequences are randomly generated from drosophila genome dm6 to match lengths of positive sequences and to not overlap them.

The *Human enhancers Ensembl* dataset was constructed from Human enhancers from The FANTOM5 project [29] accessed through the Ensembl database [47]. Negative sequences have been randomly generated from the Human genome GRCh38 to match the lengths of positive sequences and not overlap them.

The *Human non-TATA promoters* dataset was adapted from [41]. These sequences are of length 251bp: from -200 to +50bp around transcription start site (TSS). To create non-promoters sequences of length 251bp, the authors of the original paper used random fragments of human genes located after first exons.

The *Human ocr Ensembl* dataset was constructed from the Ensembl database [47]. Positive sequences are Human Open Chromatin Regions (OCRs) from The Ensembl Regulatory Build [44]. Open chromatin regions are regions of the genome that can be preferentially accessed by DNA regulatory elements because of their open chromatin structure. In the Ensembl Regulatory Build, this label is assigned to open chromatin regions, which were experimentally observed through DNase-seq, but covered by none of the other annotations (enhancer, promoter, gene, TSS, CTCF, etc.). Negative sequences were generated from the Human genome GRCh38 to match the lengths of positive sequences and not overlap them.

The *Human regulatory Ensembl* dataset was constructed from Ensembl database [47]. This dataset has three classes: enhancer, promoter and open chromatin region from The Ensembl Regulatory Build [44]. Open chromatin region sequences are the same as the positive sequences in the Human ocr Ensembl dataset.

### Reproducibility

The pre-processing and data cleaning process we followed is fully reproducible. We provide a Jupyter notebook that can be used to recreate each given dataset, and can be found in the docs folder of the GitHub repository^4^. All dependencies are provided, and a fixed random seed is set so that the notebook will always produce the same data splits.

Each dataset is divided into training and testing subsets. For some datasets, which contain only positive samples, we had to generate appropriate negative samples (dummy mouse enhancers Ensembl, drosophila enhancers stark, human enhancers Ensembl and human open chromatin region Ensembl dataset). Negative samples were selected from the same genome as the positive samples. For each positive sample, we generated a random interval in the genome with the same length as a given sample. We picked only those intervals not overlapping with any of the positive samples.

### Data format

All samples were stored as genomic coordinates, and datasets originally provided as sequences (human enhancers Cohn, human nonTATA promoters) were mapped to the reference using the ‘seq2loc‘ tool included in the package. Data were stored as compressed (gzipped) CSV tables of genomic coordinates, containing all information typically found in a BED format table. Column names are *id*, *region*, *start*, *end*, and *strand*. Each dataset has *train* and *test* subfolders and a separate table for each class. Furthermore, each dataset contains a YAML information file with metadata such as its version, the names of included classes, and links to sequence files of the reference genome. The stored coordinates and linked sequence files were used to produce the final datasets, ensuring the reproducibility of our method. For more information, visit the datasets folder of the GitHub repository^5^. To speed up this conversion from a list of genomic coordinates to a locally stored folder of nucleotide sequences, we provide a cloud based cache of the full sequence datasets which can be used simply by setting the use_cloud_cache=True option.

## Utility and discussion

### Easy data access tools

**Fig. 2:**
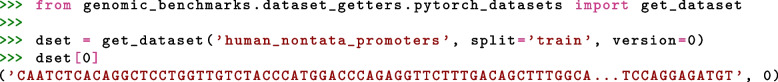
Python code for loading dataset as a PyTorch Dataset object using get_dataset() function. This function takes three arguments: name of dataset, train or test split, and version of the dataset

**Fig. 3:**
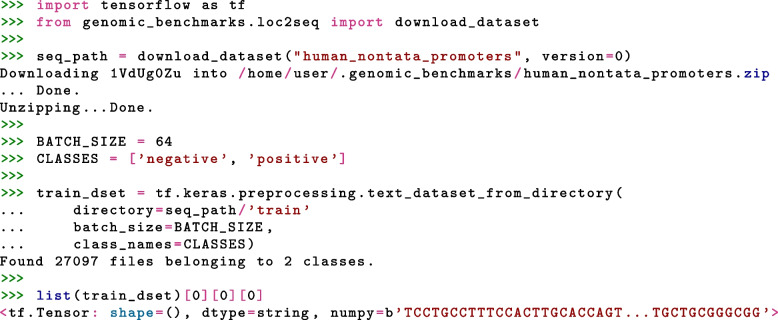
Python code for loading the dataset as TensorFlow Dataset object. First, we download dataset to our local machine and then we use TensorFlow function text_dataset_from_directory() to create a Dataset object

**Fig. 4:**
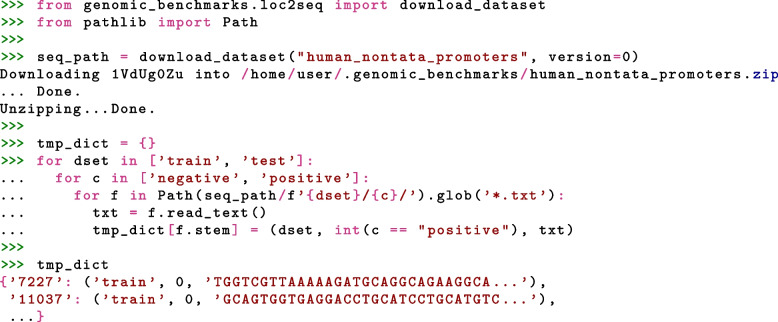
Python code for downloading and acessing the dataset as a raw text files. First, we download dataset to our local machine and then we sequentialy read all files and store the samples in a dictionary. A full example can be found at https://github.com/ML-Bioinfo-CEITEC/genomic_benchmarks/blob/main/notebooks/How_To_Train_BERT_Classifier_With_HF.ipynb

Python package with the data is installed using one command line command: pip install genomic-benchmarks. The installed package contains ready-to-use data loaders for the two most commonly used deep learning frameworks, TensorFlow and PyTorch. This feature is important for reproducibility and for the adoption of the package, particularly by people with limited knowledge of genomics. Data loaders allow the user to load any of the provided datasets using single line of code. Full examples including imports and accessing one sample of the data are shown in Figs. 2 and 3 for PyTorch and TensorFlow respectively. However, our data are not bound to any particular library or a tool. We provide an interface to the two most commonly used deep learning frameworks, but data are easily accessible using even plain Python, as shown in Fig. 4. Furthermore, we made Genomic benchmarks available as Hugging Face datasets^6^, expanding their acessibility.

### Baseline model

**Fig. 5:**
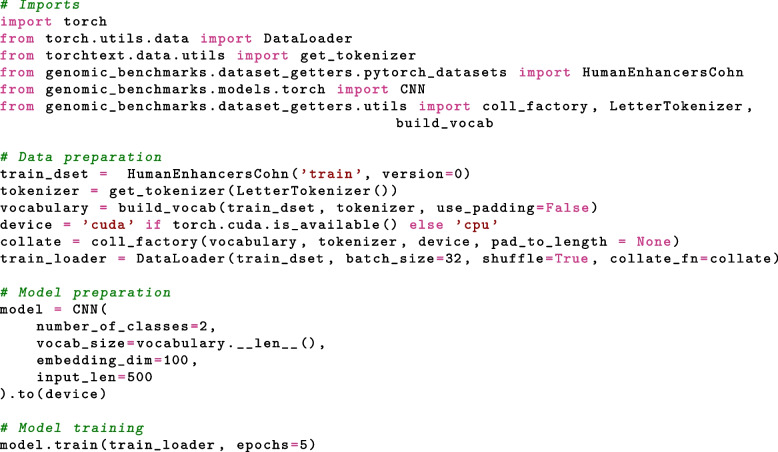
Python code showing the whole process of getting the dataset, tools, model and training the CNN model on the dataset. Thanks to out package, necessary code has only few lines and is easily understandable and expandable

**Fig. 6:**
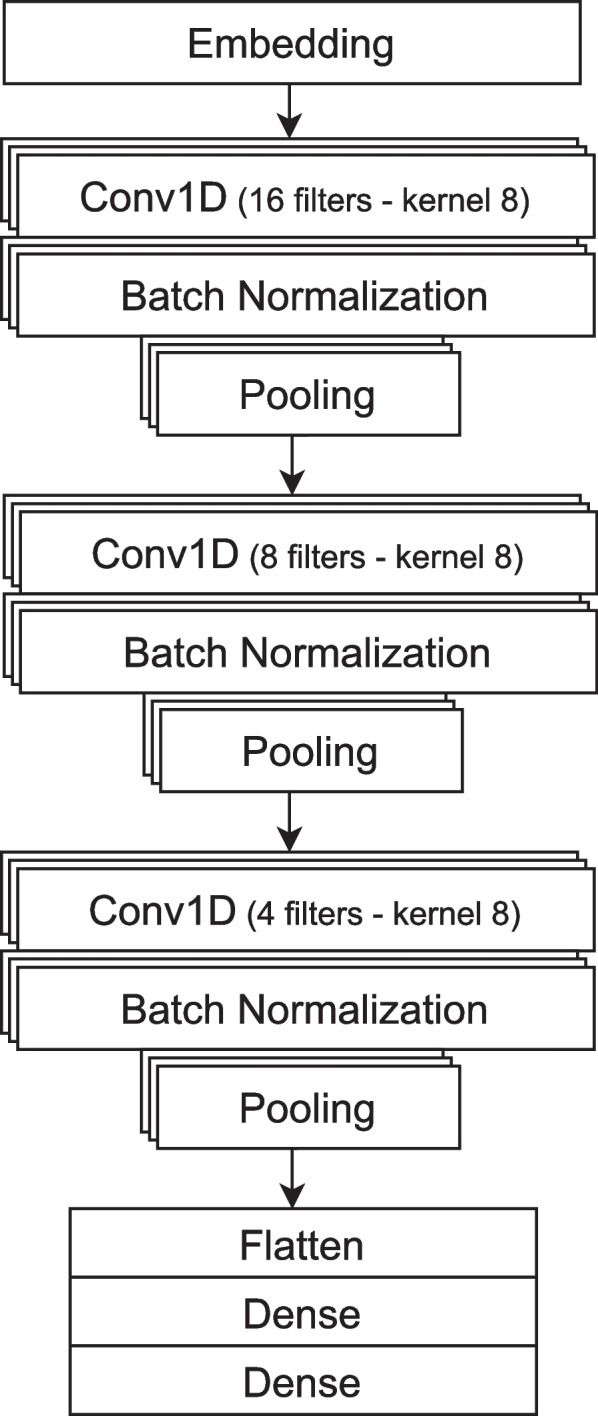
CNN architecture. The neural network consists of three convolutional layers with 16, 8, and 4 filters, with a kernel size of 8. The output of each convolutional layer goes through the batch normalization layer and the max-pooling layer. The output is then flattened and passes through two dense layers. The last layer is designed to predict the probabilities that the input sample belongs to any of the given classes

On top of ready-to-use data loaders, we provide tools for training neural networks and simple convolutional neural network (CNN) architecture (adapted from [48]). Demonstrative Jupyter notebook is provided in the notebooks folder of the GitHub repository^7^, PyTorch version is also shown in Fig. 5, and it can be used as a starting point for further research and experimentation with genomic benchmark data. CNN is an architecture that is able to find input features without feature engineering and has a relatively small number of parameters due to weights sharing (see [49] for more). Our implementation consists of three convolutional layers with 16, 8, and 4 filters, with a kernel size of 8. The output of each convolutional layer goes through the batch normalization layer and the max-pooling layer. The output of the last set of layers is flattened and goes through two dense layers. The last layer is designed to predict probabilities that the input sample belongs to any of the given classes. The architecture of the model is shown in Fig. 6. To get a baseline estimate for researchers using these benchmarks, we fit the CNN model described above to each dataset included in our collection. Training notebooks are provided in an experiments folder of the GitHub repository^8^. The models were trained for 10 epochs with batch size 64. The accuracy and F1 score for PyTorch and Tensorflow CNN models on all genomic benchmark datasets are shown in Table 2. In addition, we provide an example notebook how to train a DNABERT model [50] using Genomic Benchmarks^9^.

**Table 2:** Performance of baseline models on benchmark datasets

	Pytorch		Tensorflow	
Dataset	Accuracy	F1 score	Accuracy	F1 score
dummy_mouse_enhancers_ensembl	69.0	70.4	50.0	66.9
demo_coding_vs_intergenomic_seqs	87.6	86.8	89.6	89.4
demo_human_or_worm	93.0	92.8	94.2	93.2
drosophila_enhancers_stark	58.6	44.5	52.4	69.1
human_enhancers_cohn	69.5	67.1	68.9	71.3
human_enhancers_ensembl	68.9	56.5	81.1	74.6
human_ensembl_regulatory	93.3	93.3	79.3	79.3
human_nontata_promoters	84.6	83.7	86.5	84.4
human_ocr_ensembl	68.0	66.1	68.8	72.0

### Future development

We are aware of the limitations of the current repository. While we strive to include diverse data, still most of our benchmark datasets are balanced, or close to balanced, having similar length of sequences and a limited number of classes. Our main datasets all come from the human genome, and all deal with regulatory features. In the future, we would like to increase the diversity of our datasets to be able to diagnose the model’s sensitivity to those factors. Many machine learning tasks in Genomics consist of binary classification of a class of Genomic functional elements against a background. However, it can be beneficial to start expanding the field into multi-class classification problems, especially for functional elements that have similar characteristics to each other against the background. We will expand our benchmark collection to include more imbalanced datasets, and more multi-class datasets.

## Conclusions

Machine learning, especially deep learning, have recently started revolutionizing the field of genomics. Deep learning methods are highly dependent on large amounts of high-quality data to train and benchmark data are needed to accurately compare performance of different models. Here, we propose a collection of Genomic Benchmarks, produced with the aim of being easily accessible and reproducible. Our intention is to lower the difficulty of entry into the machine learning for Genomics field for researchers that may not have extensive knowledge of Genomics but want to apply their knowledge of machine learning in this field. Such an approach worked well for the field of protein folding, where benchmark-based competitions helped revolutionize the field.

The nine genomics datasets that have been currently added are a first step towards the direction of a large repository of Genomic Benchmarks. Beyond making access to these datasets easy for users, we have ensured that adding more datasets in a reproducible way is an easy task for further development of the repository. We encourage users to propose datasets or subfields of interest that would be useful in future releases. We have provided guidelines and tools to unify access to any genomic data and we will happily host submitted genomic datasets of sufficient quality and interest.

In this manuscript, we have implemented a simple convolutional neural network as a baseline model trained and evaluated on all of our datasets. Improvement on this baseline will be certainly achieved by using different architectures and training schemes. We have an open call for users that outperform the baseline to submit their solution via our Github repository, and be added to a ’Leaderboard’ of methods for each dataset. We hope that this will create a healthy competition on this set of reproducible datasets, and promote machine learning research in Genomics.

## Data Availability

The datasets generated and/or analysed during the current study are available in the GitHub repository, https://github.com/ML-Bioinfo-CEITEC/genomic_benchmarks.

## Abbreviations

CNN: Convolutional neural network
OCR: Open chromatin region
TSS: Transcription start site

## Footnotes

1 https://github.com/ML-Bioinfo-CEITEC/genomic_benchmarks

2 https://pypi.org/project/genomic-benchmarks/

3 https://github.com/konstantint/pyliftover

4 https://github.com/ML-Bioinfo-CEITEC/genomic_benchmarks/tree/main/docs

5 https://github.com/ML-Bioinfo-CEITEC/genomic_benchmarks/tree/main/datasets

6 https://huggingface.co/katarinagresova

7 https://github.com/ML-Bioinfo-CEITEC/genomic_benchmarks/tree/main/notebooks

8 https://github.com/ML-Bioinfo-CEITEC/genomic_benchmarks/tree/main/experiments

9 https://github.com/ML-Bioinfo-CEITEC/genomic_benchmarks/blob/main/notebooks/How_To_Train_BERT_Classifier_With_HF.ipynb
